# When homoplasy mimics hybridization: a case study of Cape hakes (*Merluccius capensis* and *M. paradoxus*)

**DOI:** 10.7717/peerj.1827

**Published:** 2016-03-28

**Authors:** Romina Henriques, Sophie von der Heyden, Conrad A. Matthee

**Affiliations:** Evolutionary Genomics Group, Department of Botany and Zoology, Stellenbosch University, Stellenbosch, South Africa

**Keywords:** Homoplasy, Hybridization, Microsatellite markers, *Merluccius*

## Abstract

In the marine environment, an increasing number of studies have documented introgression and hybridization using genetic markers. Hybridization appears to occur preferentially between sister-species, with the probability of introgression decreasing with an increase in evolutionary divergence. Exceptions to this pattern were reported for the Cape hakes (*Merluccius capensis* and *M. paradoxus*), two distantly related Merluciidae species that diverged 3–4.2 million years ago. Yet, it is expected that contemporary hybridization between such divergent species would result in reduced hybrid fitness. We analysed 1,137 hake individuals using nine microsatellite markers and control region mtDNA data to assess the validity of the described hybridization event. To distinguish between interbreeding, ancestral polymorphism and homplasy we sequenced the flanking region of the most divergent microsatellite marker. Simulation and empirical analyses showed that hybrid identification significantly varied with the number of markers, model and approach used. Phylogenetic analyses based on the sequences of the flanking region of Mmerhk-3b, combined with the absence of mito-nuclear discordance, suggest that previously reported hybridization between *M. paradoxus* and *M. capensis* cannot be substantiated. Our findings highlight the need to conduct *a priori* simulation studies to establish the suitability of a particular set of microsatellite loci for detecting multiple hybridization events. In our example, the identification of hybrids was severely influenced by the number of loci and their variability, as well as the different models employed. More importantly, we provide quantifiable evidence showing that homoplasy mimics the effects of heterospecific crossings which can lead to the incorrect identification of hybridization.

## Introduction

One of the main aims of the field of evolutionary biology is to investigate and ultimately understand the mechanisms and processes underlying the origin and evolution of species. The classical concept of reproductive isolation as an essential part for biological speciation has increasingly been challenged, since multiple examples are known of speciation with gene flow (Feder, Egan & Nosil, 2012; Martin et al., 2013; Nosil, 2008). In fact, hybridization and introgression significantly influence the evolutionary history of species, and these processes are often linked to the onset of radiation and isolation events (Barton, 2001; Joyce et al., 2011; Mallet, 2005). It is estimated that 10–25% of terrestrial and freshwater taxa can hybridize and produce viable offspring (Mallet, 2005). Thus, hybridization and introgression are no longer perceived as evolutionary dead-ends, but rather as potential sources of variation (Mallet, 2005).

For most marine taxonomic groups, information regarding the occurrence, frequency and viability of natural hybrids is sparse compared to terrestrial systems (Montanari et al., 2012). Until recently, hybridization was thought to be uncommon and confined to a few taxa in narrow hybrid zones (Rao & Lakshmi, 1999). However, advances in molecular techniques, combined with more comprehensive sampling efforts, and the development of individual-based assignment tests are contributing to a shift in the field. In the last decade, hybridization in the marine environment moved from rare (Roques, Sevigny & Bernatchez, 2001) to being considered an important mechanism in radiation events and speciation of multiple taxa (Bowen et al., 2013; Litsios & Salamin, 2014). A brief literature review reveals that hybrids are commonly found between species of mussels (Bierne et al., 2003), fishes (Albert, Jonsson & Bernatchez, 2006; Potts et al., 2014), turtles (Vilaca et al., 2012), sea snakes (Sanders, Rasmussen & Guinea, 2014) and marine mammals (Attard et al., 2012), where two major trends can be identified: 63% of all taxa that hybridize are sister-species and 73% occur in sympatry (for full reference list see Table S1). Although hybridization can also occur between distantly related species (Bernardi et al., 2013; Gaither et al., 2014; Garret et al., 2007), several studies have highlighted the link between genetic distance (perceived as evolutionary divergence) and potential for hybridization, since closely related species are more likely to have similar biological, ecological and behavioural features that can increase the frequency of heterospecific crossings (Edmands, 2002; Mallet, 2005; Montanari et al., 2014; Montanari et al., 2012). In addition, the success and viability of hybrids may decrease with higher divergence due to the establishment of genetic incompatibilities (Abbott et al., 2013). Genetic distances thus appear to be good predictors for the frequency and success of natural hybridization events (Edmands, 2002; Mallet, 2005). In terrestrial/freshwater systems, a genetic distance between 0.05–0.10 (based on uncorrected pairwise differences, *p*, for coding regions of the mitochondrial DNA) is generally considered to be an important threshold after which hybrids are no longer viable (Mallet, 2005). However, for marine species the cut-off point appears to be lower (*p* = 0.04–0.05), and very few hybridization cases have been reported between highly divergent species. Exceptions to this are: *Chrysoblephus anglicus* x *C. puniceus* (*p* = 0.12—Von der Heyden & Connell, 2012); *Siganus corallinus* x *S. puellus* (*p* = 0.1—Kuriiwa et al., 2007); *Solea senegalensis* x *S. aegyptiaca* (*p* = 0.086—Ouanes et al., 2011); *Merluccius albidus* x *M. billinearis* (*p* = 0.077—Machado-Schiaffino, Juanes & Garcia-Vazquez, 2010) and *M. paradoxus* x *M. capensis* (*p* = 0.077—Miralles, Machado-Schiaffino & Garcia-Vazquez, 2014). In the first two examples, the authors found low levels of hybridization and all hybrids were identified as first generation (*F*_1_), questioning the long-term viability and persistence of such crossings. In contrast, for the *Solea* and *Merluccius* cases, a high frequency of hybrids was reported (36%, 4.3–28% and 17%, respectively), with evidence of multiple introgression events (Machado-Schiaffino, Juanes & Garcia-Vazquez, 2010; Miralles, Machado-Schiaffino & Garcia-Vazquez, 2014; Ouanes et al., 2011). The latter findings thus suggest that hybridization in these species is a common event and spans multiple generations, despite high genetic divergence and, in the case of the hakes, also the absence of a sister-species relationship of the taxa involved.

The genus *Merluccius* (hakes) comprises 16 offshore demersal species occurring throughout the Atlantic and the Pacific Oceans (Froese & Pauly, 2014). All species are important commercial fishery resources, and the majority are currently considered over-exploited (FAO, 2014). Contrary to the example of *S. senegalensis* and *S. aegyptiaca*, the *Merluccius* taxa reported to hybridize are not sister-species, and isolation is estimated to have occurred around 3-4.2 Million years ago (Ma) (Campo et al., 2007; Grant & Leslie, 2001; Quinteiro, Vidal & Rey-Mendez, 2000). In particular, *M. paradoxus* and *M. capensis,* although partially sympatric, have colonized the southeastern Atlantic in two independent events (Campo et al., 2007; Grant & Leslie, 2001). Adults occupy different depths and ecotypes, with overlap in distribution confined primarily to the early life-stages (Botha, 1985; Von der Heyden, Lipinski & Matthee, 2007b). Furthermore, recent studies suggest that while *M. capensis* exhibits two spawning grounds in the region, one off of central Namibia and one off of the West Coast of South Africa, there is no evidence for the presence of spawning *M. paradoxus* adults off Namibia, with spawning grounds confined to South Africa (Jansen et al., 2015; Stromme, Lipinski & Kainge, 2015). These would theoretically minimise the possibility of regular hybridization events, and decrease the probability of a north-south hybridization cline as reported in Miralles, Machado-Schiaffino & Garcia-Vazquez (2014). Therefore, the evolutionary distance and life history of *M. paradoxus* and *M. capensis*, suggests that they are not likely candidates for the occurrence of multiple hybridization and introgression events. This hypothesis is also supported by a previous study using allozymes, which indicated complete reproductive isolation between the two species (Grant & Leslie, 2001).

Interestingly, while the identification of hybrids in *Chrysoblephus*, *Siganus* and *Solea* was conducted using nuclear gene sequences and/or allozymes, both reports of hybridization in *Merluccius* (Machado-Schiaffino, Juanes & Garcia-Vazquez, 2010; Miralles, Machado-Schiaffino & Garcia-Vazquez, 2014) relied on five and six cross-specific nuclear microsatellite markers (respectively) and mitochondrial DNA (mtDNA). Since nuclear microsatellites are one of the most variable types of genetic markers, with high levels of heterozygosity they are frequently used to infer gene exchange in population genetic studies (Ellegren, 2004; Selkoe & Toonen, 2006). The high level of variation, however, makes them prone for accumulating homoplasy (Angers & Bernatchez, 1997; Ellegren, 2004; Estoup, Jarne & Cornuet, 2002; Grimaldi & CrouauRoy, 1997; Van Oppen et al., 2000).

In hybridization studies based on microsatellite markers, the identification of individuals of admixed origins relies on the existence of different allelic profiles when compared to the parents’ populations/species. It is, however, necessary to distinguish among the three possible sources of this variation: interbreeding, incomplete lineage sorting and homoplasy. Although there have been extensive studies cautioning the use of microsatellite markers in admixture analyses (Angers & Bernatchez, 1997; Ellegren, 2004; Estoup, Jarne & Cornuet, 2002; Grimaldi & CrouauRoy, 1997; Van Oppen et al., 2000), the majority of population genetic and hybridization studies in the marine environment did not explicitly account for the presence of homoplasy. In particular, Van Oppen et al. (2000) revealed that homoplasy can quickly accumulate even between recently diverged species. This suggests that microsatellite markers might not be suitable for describing hybridization events between Cape hakes due to species’ highly divergent evolutionary history spanning millions of years.

In order to resolve the apparent hybridization conundrum, we extend previous work (Miralles, Machado-Schiaffino & Garcia-Vazquez, 2014) by employing extensive sampling strategy across the species distribution ranges and analysing each individual using a combination of microsatellite loci and the control region (CR) of mitochondrial DNA (mtDNA). Here, we aim to assess the validity of the previous results of hybridization (Miralles, Machado-Schiaffino & Garcia-Vazquez, 2014). Specifically, the aims of this study are: (a) to perform *a priori* simulation analyses to establish if microsatellite loci can adequately distinguish between *M. paradoxus* and *M. capensis*, their putative hybrids and hybrid states (*F*_1_, *F*_2_, and backcrosses to both species); (b) to assess the presence and frequency of hybrids between *M. paradoxus* and *M. capensis*; and finally (c) to distinguish between interbreeding, incomplete lineage sorting and homoplasy in the assignment of putative hybrids. The latter was achieved by sequencing the flanking region of microsatellites in order to discriminate between alleles that are equal by descent or equal due to convergence. Our findings add to the growing body of literature outlining the conditions under which microsatellite markers could be used for hybridization studies. In particular, we show that without explicitly testing for homoplasy, microsatellites are not reliable for detecting hybridization between divergent species.

## Material and Methods

### Sampling

**Figure 1:**
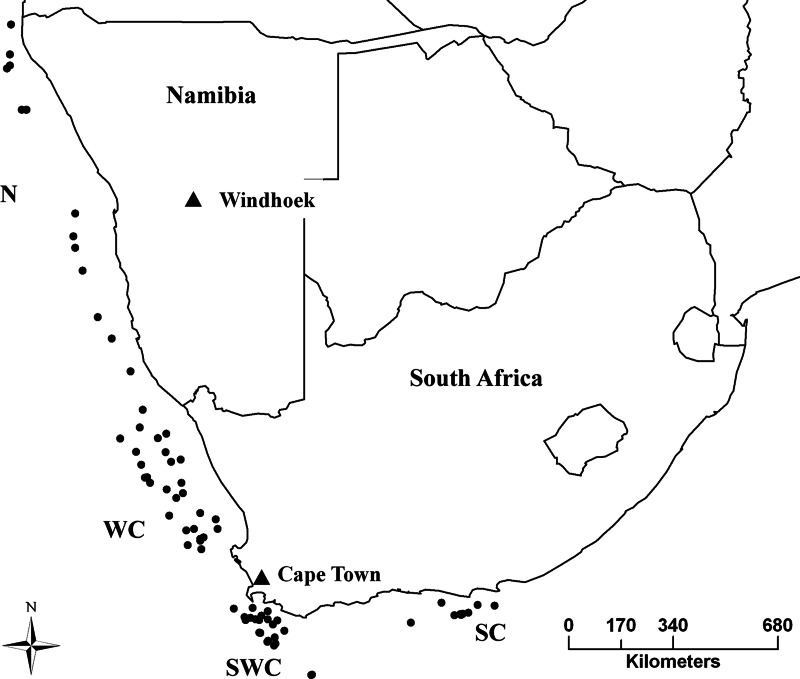
Sampling strategy and effort for *M. capensis* and *M. paradoxus*. Sampling strategy for *M. capensis* and *M. paradoxus* across Southern Africa: N, Namibia (*N* = 413); W, West Coast (*N* = 302); SW, Southwest Coast (*N* = 288); S, South Coast (*N* = 160).

Sampling took place in the years 2012–2013, from the Cunene River mouth in northern Namibia, to Port Elizabeth in South Africa, covering the entire distribution range of both species (Fig. 1). Samples were obtained from Namibian and South African governmental research surveys and commercial fisheries by trawling. A piece of muscle was collected immediately after capture and stored in 95% ethanol. DNA was extracted using a standard chlorophorm:isopropanol method (Winnepenninckx, Backeljau & Dewachter, 1993).

### Preliminary data analyses

DNA of 1,200 individuals (300 per year and per species) was Polymerase Chain Reaction (PCR) amplified for a fragment of the mitochondrial DNA control region (CR—Quinteiro, Vidal & Rey-Mendez, 2000), and for ten nuclear microsatellite loci. Although more loci should ideally be used (Cornuet et al., 1999; Vähä & Primmer, 2006; Väli et al., 2010) empirical studies suggest that 100% of correct assignments can be achieved with ten loci, as long as they have an average genetic diversity of H ∼ 0.6 and 30–50 individuals per population are analysed (Cornuet et al., 1999). Moreover, the usage of ten loci allows for direct comparison with previous published work on hybridization in the marine environment (Table S1), and in particular Miralles, Machado-Schiaffino & Garcia-Vazquez (2014). Seven newly developed for *M. paradoxus* (MP51, MP318, MP374, MP8450, MP8478, MP8494, MP8448—Hoareau et al., 2015) and three developed for *M. merluccius* also used in the previous hybridization study (Mmerhk-3b, Mmerhk-20, Mmerhk-29—Moran et al., 1999).

All PCR methods followed the original protocols. The mtDNA PCR products were sequenced (CAF, Stellenbosch, South Africa) and final alignment was conducted using CLUSTAL X (Thompson et al., 1997) in GENEIOUS 7.1.4 (Biomatters, Auckland, New Zealand). Microsatellite fragments were genotyped in an ABI377 (CAF, Stellenbosch, South Africa) using LIZ as an internal size marker, and scored based on size in GENEIOUS 7.1.4. Accuracy of scores was ensured by using a reference individual as positive control. The microsatellite dataset was evaluated for quality of amplification by estimating the occurrence of null alleles, large allele drop out and stuttering in MICROCHECKER (Van Oosterhout, Weetman & Hutchinson, 2006). Deviations to the expectation of outcrossing and linkage disequilibrium were tested in FSTAT (Goudet, 1995). Statistical significance was tested with 10,000 permutations. One locus was found to be in linkage with locus MP374, and the latter was therefore removed from further analyses. The possibility of temporal differentiation was tested by performing pairwise genetic divergence analyses between sampling years for both species for the CR dataset in ARLEQUIN (Excoffier, Laval & Schneider, 2005), and for the nine microsatellite loci in FreeNA (Chapuis & Estoup, 2007), with statistical significance assessed after 10,000 iterations. As no evidence of genetic differentiation was observed, sampling years were pooled together and treated as one single population. Genetic diversity levels were measured as haplotype (h) and nucleotide (*π*) diversity for mtDNA in ARLEQUIN, and expected (*H*_*E*_) and observed heterozygosity (*H*_*O*_), number of alleles (Na), allelic richness (AR), percentage of null alleles (NAL) and inbreeding index (*F*_IS_) for the microsatellite dataset in ARLEQUIN, FSTAT and FreeNA. Deviations of neutral expectations were tested within geographical regions (Namibia vs. South Africa) and between species (*M. capensis* vs. *M. paradoxus*) using the *F*_ST_-based approach implemented in LOSITAN (Antao et al., 2008), under the Stepwise Mutation Model (SMM) and run for 50,000 simulations.

The ability of the microsatellite dataset to distinguish between *M. paradoxus* and *M. capensis* was investigated by: (a) constructing a distribution of allelic frequencies, to assess the presence and frequency of fixed alleles, (b) estimating pairwise *F*_ST_ values in FreeNA (Chapuis & Estoup, 2007), with statistical significance assessed after 10,000 iterations, and (c) performing a Principal Component Analysis (PCA) based on allelic frequencies in adegenet 1.3.1 (Jombart & Ahmed, 2011; this method is less influenced by deviations to Hardy-Weinberg equilibrium and the presence of null alleles). Furthermore, as different age classes of both species are commonly caught together and external morphological identification is difficult, preliminary analyses of individual assignment were conducted using both mtDNA and nDNA datasets to validate morphological identification. Medium-joining networks were reconstructed for the CR sequences of both species combined in NETWORK (Bandelt, Forster & Rohl, 1999). The coalescent-based assignment software STRUCTURE (Pritchard, Stephens & Donnelly, 2000) was used to assign individuals to species, based on microsatellite data. Five preliminary runs for *K* = 2 were performed under a strict assignment model, with no-admixture and independent allelic frequencies, for 250,000 MCMC iterations, after an initial burnin of 50,000 MCMC steps. The five runs were combined in CLUMPP (Jakobsson & Rosenberg, 2007), and final results visualized in DISTRUCT (Rosenberg, 2004).

### Assignment analyses and identification of hybrid states

Assignment tests and identification of hybrids were performed in a two-step manner. First, a simulation study was conducted in Hybridlab (Nielsen, Bach & Kotlicki, 2006) to assess if the microsatellite dataset could accurately differentiate among multiple hybridization events, i.e. that hybrids were viable and capable of backcrossing with their parental species. Four hybrid states were simulated: first generation (*F*_1_), second generation (*F*_2_), backcrosses of F1 with *M. paradoxus* (BcP) and backcrosses of F1 with *M. capensis* (BcC). A total of 80 simulated hybrids were generated based on the allelic frequencies of 160 randomly chosen individuals identified as “pure” *M. paradoxus* and *M. capensis* in the previous STRUCTURE analysis, using a cut-off value of 0.9 for the ancestry coefficient (*q*). This simulated dataset was then used in Bayesian clustering analyses implemented in STRUCTURE and NEWHYBRIDS (Anderson & Thompson, 2002). Both approaches rely on the assessment of an ancestry coefficient (*q*) for each individual based on the allelic frequencies of the identified clusters. We ran STRUCTURE analyses for four different admixture models mimicking a range of evolutionary scenarios: (i) no interbreeding and two independently evolving species (no admixture and independent allelic frequencies); (ii) no interbreeding, but species sharing a recent common ancestor (no admixture and correlated allelic frequencies); (iii) interbreeding but two distantly related species (admixture and independent allelic frequencies) and (iv) interbreeding between recently evolved species/populations (admixture and correlated allelic frequencies) (Pritchard, Stephens & Donnelly, 2000). Each model was assessed with five preliminary runs, for *K* = 2, with an initial burnin of 50000 MCMC steps, followed by 250000 MCMC iterations. The five runs were combined in CLUMPP, and results visualized in DISTRUCT. There is no general consensus regarding the most likely *q* to distinguish between “pure” and admixed individuals, with the choice of *q* greatly depending on the purpose of the study (Vähä & Primmer, 2006). A *q* = 0.2 (20% of admixture) increases accuracy: the ability to correctly assign individuals to their classes (“pure” vs hybrids), while *q* = 0.1 (10% of admixture) will decrease the probability of incorrectly assigning “pure” as hybrids (Vähä & Primmer, 2006). As one of the main aims of this study is to detect admixed individuals and assess if the microsatellite dataset can accurately distinguish between different hybrid states, we employed both *q* levels to understand how this metric influences the number of putative hybrids identified (Bohling, Adams & Waits, 2013; Vähä & Primmer, 2006; Väli et al., 2010). Only individuals with non-overlapping credibility intervals were retained as putative hybrids. Finally, identification of hybrids states was conducted in NEWHYBRIDS. Five runs were performed with different initial seed values, using a Jeffrey prior, for 250,000 MCMC iterations, after an initial burnin of 50,000 MCMC steps. Runs were combined in CLUMP and individuals identified based on *q* = 0.5 (Anderson & Thompson, 2002). The accuracy of both approaches was recorded as a percentage of correct assignments to each state. Once the simulation study was performed, the same analyses were conducted for the true microsatellite dataset.

Preliminary analyses revealed significant deviations to the expectations of outcrossing, mainly in *M. capensis*, the presence of null alleles for both species and deviations from neutrality in three loci (MP318, MP8450 and Mmerhk-3b). Although these assignment tests are generally robust and can be performed even in non-ideal conditions (Carlsson, 2008; Cornuet et al., 1999; Hauser et al., 2006), we created two additional sub-datasets: (a) removing loci with a null allele frequency above 15% and (b) without the three loci under selection, in order to understand how these features influence the identification of putative hybrids in the Cape hakes. Both datasets were run in STRUCTURE (only with model iii—admixture with independent allelic frequencies for computational purposes) and NEWHYBRIDS, with the same run conditions described above.

### Verification of hybrids

A total of 38 “pure” individuals and 19 putative hybrids identified with more than one assignment method were further PCR amplified and sequenced for Mmer-hk3b, following the original protocol (Moran et al., 1999). At the microsatellite level, the sequenced hybrids were either heterozygous, exhibiting allele sizes from both species (e.g., 327/341—*M. capensis*/*M. paradoxus*), or homozygous for the alleles of the other species (*M. capensis*: 341/341; *M. paradoxus*: 327/327). Furthermore, individuals with mito-nuclear discordance were re-sequenced for mtDNA, to confirm their status. The obtained sequences were aligned using CLUSTAL X, in GENEIOUS 1.7.4, and used for hybrid identification. Individuals with admixed origin are expected to be heterozygous for sites with species-specific mutations, and alleles of F1 individuals should thus appear in both species specific clades after a gene tree is constructed for this data. By comparing direct sequences of the flanking regions, it is also possible to identify potential homoplasy due to insertions or deletions either in the core sequence of the microsatellite, or in the flanking region (Estoup, Jarne & Cornuet, 2002; Grimaldi & CrouauRoy, 1997; Van Oppen et al., 2000). In order to understand if admixed individuals resulted from interbreeding, incomplete lineage sorting or homoplasy, we used the available sequence of Mmerhk-3b for *M. merluccius* in Genbank (Accession number: AF136627.1) in the phylogenetic analyses. Reconstruction of phylogenetic relationships was performed with the Maximum Likelihood algorithm implemented in PhyML (Guindon et al., 2009), using the most suitable nucleotide substitution model as indicated by jModelTest (Posada, 2008). Nodal support was obtained employing the *X*^2^-arLT statistics (Anisimova & Gascuel, 2006), and trees visualized in Figtree (Rambaut, 2009).

## Results

### Preliminary analyses

Of 1,181 individuals, 1,137 were successfully PCR amplified and sequenced/genotyped for both mtDNA and the microsatellite datasets. As extensive introgression (via mito-nuclear discordance) was described in Miralles, Machado-Schiaffino & Garcia-Vazquez (2014), only individuals amplified for both datasets were retained for further analyses.

Assessment of amplification quality of microsatellite loci revealed that although there was no evidence of large allele drop out or stuttering, both species’ datasets contained null alleles and did not conform to Hardy-Weinberg expectations, due to heterozygotes deficit (Table 2). Overall, *M. paradoxus* had a lower frequency of null alleles (∼3%) at the cross-specific loci Mmerhk-20 and Mmerhk-29, and at locus MP8450. In contrast, the null allele frequencies in *M. capensis* ranged from 1–22.9% across six loci (Table 2). Locus MP8448 and Locus MP374 were discovered to be in linkage, and thus the first was removed from the final dataset. All subsequent analyses were performed with nine microsatellite loci. Neutrality tests performed within and between species revealed outlier markers, which appeared to be under positive selection. Loci MP318, MP8450 and Mmerhk-3b were flagged as outlier loci between *M. paradoxus* and *M. capensis* (*F*_ST_ > 0.1, *p* < 0.05). Loci MP8894 and Mmerhk-3b were revealed to be non-neutral in *M. capensis* (*F*_ST_ > 0.1, *p* < 0.05). No significant deviations to neutrality were observed for the *M. paradoxus* dataset.

**Table 1:** Genetic diversity measures at nine microsatellite loci for *M. capensis* and *M. paradoxus* (both sampling years combined).

		*M. capensis*			*M. paradoxus*		
		Namibia	South Africa	Total	Namibia	South Africa	Total
MP318	*n*	190	390	580	342	214	566
	*Na*	7	8	10	14	12	14
	*NAL*	8.78	7.67	8.05	2.42	1.05	1.44
	*AR*	6.368	6.1	9.457	12.917	11.289	13.92
	*H*_*E*_	0.254	0.286	0.275	0.732	0.741	0.737
	*H*_*O*_	0.179	0.221	0.207	0.678	0.740	0.716
	*F*_IS_	**0.296**	**0.228**	**0.248**	0.075	0.001	0.029
MP8748	*n*	188	388	576	212	340	552
	*Na*	29	31	33	19	24	27
	*NAL*	2.95	1.03	1.64	0.09	1.73	0.99
	*AR*	26.425	27.71	32.456	17.566	18.682	26.536
	*H*_*E*_	0.863	0.874	0.870	0.867	0.869	0.868
	*H*_*O*_	0.803	0.869	0.847	0.892	0.832	0.855
	*F*_IS_	**0.069**	0.006	**0.027**	−0.028	0.042	0.015
MP51	*n*	190	389	579	215	342	557
	*Na*	9	11	13	12	12	14
	*NAL*	0.00	0.01	0.19	0.28	0.06	0.16
	*AR*	8.693	8.577	12.56	10.646	11.338	13.779
	*H*_*E*_	0.349	0.286	0.307	0.556	0.508	0.526
	*H*_*O*_	0.363	0.288	0.313	0.558	0.535	0.544
	*F*_IS_	−0.042	−0.008	−0.019	−0.004	−0.054	−0.034
MP8894	*n*	150	367	517	202	324	526
	*Na*	8	5	8	18	19	22
	*NAL*	23.76	10.27	22.86	0.00	1.71	1.13
	*AR*	6.539	5	8	15.953	16.131	21.914
	*H*_*E*_	0.498	0.299	0.540	0.631	0.672	0.657
	*H*_*O*_	0.173	0.207	0.197	0.649	0.654	0.652
	*F*_IS_	**0.308**	**0.652**	**0.635**	−0.028	0.027	0.007
MP374	*n*	189	376	565	214	342	556
	*Na*	4	5	5	5	4	5
	*NAL*	0.85	9.21	6.93	2.71	0.00	0.01
	*AR*	3.587	4.266	4.993	4.969	3.901	5
	*H*_*E*_	0.349	0.172	0.240	0.33	0.361	0.349
	*H*_*O*_	0.339	0.112	0.188	0.299	0.383	0.351
	*F*_IS_	0.03	**0.352**	**0.217**	0.094	−0.06	−0.004
MP8450	*n*	175	351	526	210	330	540
	*Na*	22	27	28	38	45	45
	*NAL*	4.11	3.34	3.62	5.31	3.31	4.13
	*AR*	21.671	23.323	27.931	36.895	38.309	44.744
	*H*_*E*_	0.892	0.911	0.906	0.946	0.951	0.949
	*H*_*O*_	0.806	0.855	0.838	0.848	0.894	0.876
	*F*_IS_	0.097	0.062	**0.074**	**0.104**	**0.06**	**0.077**
Mmerhk-20	*n*	190	387	577	214	338	552
	*Na*	26	25	27	23	24	39
	*NAL*	0	0.99	0.59	5.29	1.79	3.143
	*AR*	22.646	25.01	26.676	30.249	31.491	38.611
	*H*_*E*_	0.912	0.905	0.908	0.91	0.908	0.908
	*H*_*O*_	0.916	0.879	0.891	0.794	0.864	0.837
	*F*_IS_	0.029	−0.004	0.019	**0.127**	**0.048**	0.079
Mmerhk-29	*n*	189	376	565	214	336	550
	*Na*	25	29	30	23	24	27
	*NAL*	5.25	7.87	7.00	1.48	3.40	2.78
	*AR*	24.602	24.235	29.716	20.591	20.886	26.742
	*H*_*E*_	0.91	0.912	0.911	0.897	0.89	0.893
	*H*_*O*_	0.804	0.755	0.772	0.860	0.827	0.84
	*F*_IS_	**0.172**	**0.117**	**0.153**	0.042	0.071	0.06
Mmerhk-3b	*n*	188	380	568	207	327	534
	*Na*	8	9	10	8	8	9
	*NAL*	3.40	3.41	11.92	4.10	1.45	2.72
	*AR*	7.596	6.527	9.811	7.707	7.054	8.968
	*H*_*E*_	0.417	0.463	0.614	0.312	0.328	0.322
	*H*_*O*_	0.388	0.418	0.408	0.304	0.327	0.318
	*F*_IS_	0.096	**0.068**	**0.335**	0.003	**0.026**	**0.011**

**Notes.**

*n* Number of individuals genotyped Na Number of alleles NAL % of null alleles AR Allelic richness for a minimum of 150/517 (per region/total) individuals *H*_*E*_ Expected heterozygosity *H*_*O*_ Observed heterozygosity *F*_IS_ Inbreeding coefficient (significant deviations to Hardy-Weinberg expectations in bold, *p* < 0.05)

A fragment of 406 bp was sequenced for the CR, yielding ten haplotypes in *M. paradoxus*, and 160 haplotypes in *M. capensis*. Genetic diversity levels varied with species and dataset used (Tables 1 and 2). Overall, *M. paradoxus* had lower genetic diversity for the mtDNA and higher for the microsatellite dataset: *h* = 0.541, *π* = 0.002, *H*_*O*_ = 0.689; while *M. capensis* showed an inverse pattern with higher genetic diversity observed for the mtDNA dataset and lower for the microsatellite loci *h* = 0.893, *π* = 0.008, *H*_*O*_ = 0.619 (Tables 1 and 2). Number of alleles ranged from 5–45 per locus in *M. paradoxus* and 5–33 per locus in *M. capensis* (Table 2). No significant genetic differentiation was observed between sampling years for either dataset or species (*M. paradoxus*: *φ*_ST_ = 0.002, *F*_ST_ = 0.000, *p* > 0.05; *M. capensis*: *φ*_ST_ = 0.001, *F*_ST_ = 0.003, *p* > 0.05), and the presence of null alleles did not significantly influence differentiation measures in *M. capensis* (uncorrected *F*_ST_ = 0.0034, corrected *F*_ST_ = 0.0032). Thus, both sampling years were pooled.

**Table 2:** Genetic diversity measures based on the CR of mtDNA for *M. capensis* and *M. paradoxus* for both sampling years combined.

	*M. capensis*			*M. paradoxus*		
	Namibia	South Africa	Total	Namibia	South Africa	Total
*N*	185	371	556	215	343	558
*H*	57	124	160	6	12	14
*h*	0.845	0.915	0.893	0.528	0.546	0.541
*π*	0.005	0.009	0.008	0.002	0.002	0.002

**Notes.**

*N* Number of individuals *H* Number of haplotypes *h* Haplotype diversity *π* Nucleotide diversity (accession numbers: KU705901– KU707034)

**Figure 2:**
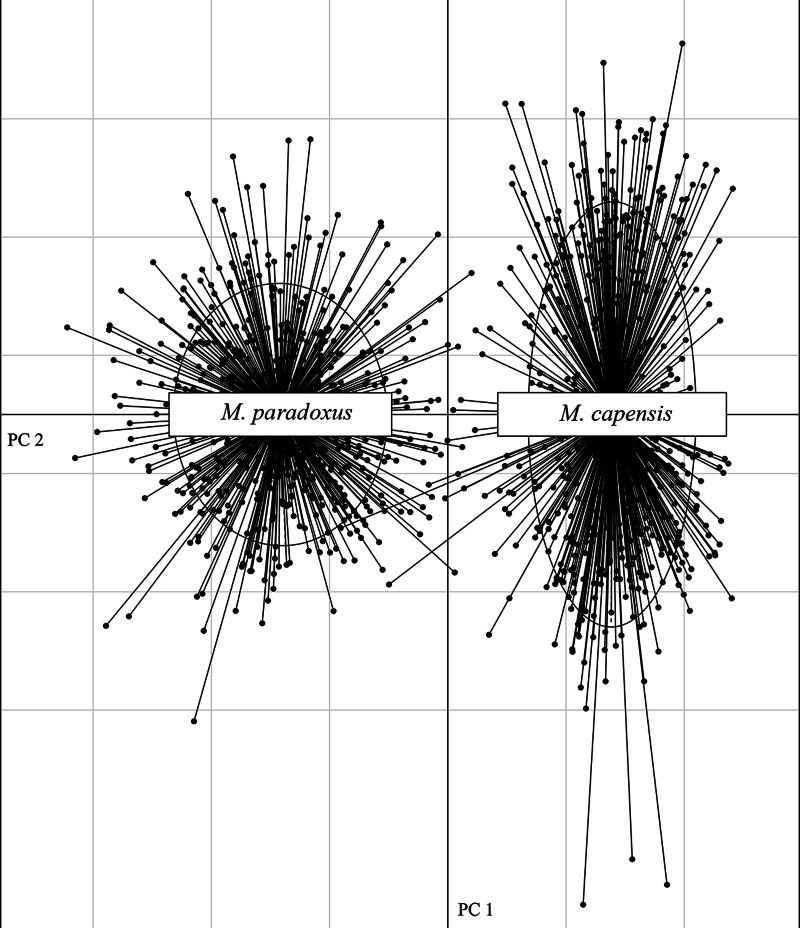
PCA figure for *M. capensis* and *M. paradoxus.* Distribution of *M. capensis* and *M. paradoxus* individuals in the two-dimensional space of a Principal Component Analysis based on allelic compositions of nine microsatellites.

The microsatellite dataset could accurately distinguish between the two species with different loci exhibiting non-overlapping allele size ranges for *M. paradoxus* and *M. capensis* (Fig. S1). In particular, locus Mmerhk-3b was the most divergent, with two sets of alleles fixed in each taxon, and only a few individuals of either species exhibiting shared alleles (Fig. S1). The PCA plot clearly separated the two species along the first two axes, with limited overlap between them (Fig. 2). Pairwise *F*_ST_ values were estimated at *F*_ST_ = 0.126 (uncorrected, *p* < 0.05) and *F*_ST_ = 0.114 (corrected, *p* < 0.05) between *M. capensis* and *M. paradoxus*.

Preliminary assignment tests using both datasets revealed the presence of 47 individuals that were misidentified during sampling (4.13% of total samples): 42 for *M. capensis* and five for *M. paradoxus*, which were reassigned to represent the correct species (Fig. S2). Reconstruction of the phylogenetic relationships between individuals clearly separated *M. capensis* and *M. paradoxus* with only two individuals identified as having mito-nuclear discordance (after correcting for sampling mislabels; Fig. S2).

### Assignment analyses and identification of hybrids

**Figure 3:**
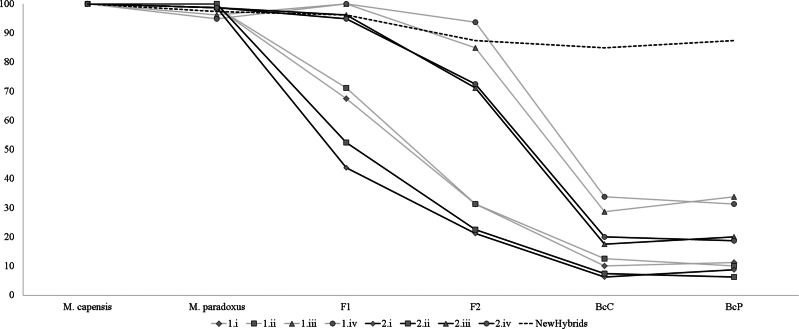
Graph depicting accuracy of assignment of “pure” and hybrid status using different models and methods. Percentage of individuals recognized as pure-bread and hybrids by multiple clustering methods and models, based on the simulated dataset obtained with Hybridlab for nine microsatellite markers. 1—*q* = 0.1 and 2—*q* = 0.2; model i (no admixture, independent allelic frequencies); model ii (no admixture, correlated allelic frequencies); model iii (admixture, independent allelic frequencies), model iv (admixture, correlated allelic frequencies).

Simulation analyses showed that identification of simulated hybrids significantly varied with the model and approach used (Fig. 3). Assignment analyses performed in STRUCTURE were able to detect F1 hybrids in the majority of cases, but accurate identification of further hybrid states varied significantly with the chosen admixture model (Fig. 3). Overall, assignment models iii and iv identified a higher number of hybrids than models i and ii, while *q* = 0.1 had a more accurate detection of multiple hybridization events (BcC and BcP) than *q* = 0.2 (Fig. 3). Successful detection of simulated F2 hybrids ranged from 21.3% (*q* = 0.2, model i) to 93.7% (*q* = 0.1, model iv); BcC hybrids ranged from 6.3% (*q* = 0.2, model i) to 33.8% (*q* = 0.1, model iv) and BcP hybrids ranged from 6.3% (*q* = 0.2, model i) to 33.8% (*q* = 0.1, model iii). However, while individuals considered “pure” had non-overlapping credibility intervals, implying that the nine microsatellites can accurately assign individuals to either species, the majority of identified hybrids exhibited overlapping intervals regardless of the method employed. The NewHybrids approach performed better and allowed to correctly identify the hybrid status in 96% of the simulated cases for F1, 87.5% for F2, 85% for BcC and 87.5% for BcP (Fig. 3).

**Figure 4:**
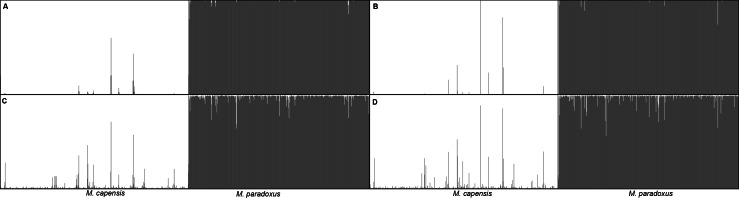
Bayesian assignment plot for *M. capensis* and *M. paradoxus*. Bayesian clustering analyses for *M. capensis* and *M. paradoxus*, using STRUCTURE and based on nine microsatellite markers: (A)—model i (no admixture, independent allelic frequencies); (B)—model ii (no admixture, correlated allelic frequencies); (C)—model iii (admixture, independent allelic frequencies), (D)—model iv (admixture, correlated allelic frequencies).

Similarly, analyses of the complete dataset revealed that the number of putative hybrids identified varied significantly with the admixture model used and the ancestry coefficient threshold (Table 3 and Fig. 4). As expected, *q* = 0.1 identified a higher number of admixed individuals when compared to *q* = 0.2: 46 vs. 16 (Table 3). Assignment models i and ii (no admixture) identified up to 14 putative hybrids (1.14%), while models iii and iv (admixture) retrieved up to 42 hybrids (3.78%) (Table 3 and Fig. 4). The NEWHYBRIDS approach identified 21 individuals as F2 (1.93%), one *M. capensis* as pure *M. paradoxus* and the remaining individuals as pure species (Table 3). Only three individuals were identified as putative hybrids by all models and approaches (0.27%), 19 (1.67%) by two or more models and of these only six had non-overlapping Bayesian credibility intervals (Table 3). The majority of admixed individuals were *M. capensis* (26), and all hybrids were found throughout the distribution range of the species, from northern Namibia to the East Coast in South Africa (Table 3).

**Table 3:** *Merluccius capensis* and *M. paradoxus* individuals identified as hybrids by multiple clustering methods and models based on nine microsatellite loci.

		Structure								
Individual	mtDNA	*q* = 0.1				*q* = 0.2				NewHybrids (*q* < 0.5)
		i	ii	iii	iv	i	ii	iii	iv	
**12_C4aS_4**	**C**		**x**	**x**	**x**					**F2**
**12_C4aS_10**	**C**		**x**	**x**	**x**			**x**	**x**	**F2**
**12_C9S_12**	**C**		**x**	**x**	**x**		**x**	**x**	**x**	**F2**
**12_C9S_16**	**C**			**x**	**x**			**x**	**x**	**F2**
12_C1SW_13^*^	C			x	x					C
**12_C1SW_15**	**C**			**x**	**x**			**x**		**F2**
**12_C1SW_21**	**C**			**x**	**x**			**x**		**F2**
12_C11SW_19	C				x					F2
12_C102W_3	C			x	x					C
12_C4N_1	C	x								C
13_C1S_277	C				x					C
**13_C8S_230**	**C**	**x**	**x**	**x**	**x**	**x**	**x**	**x**	**x**	**F2**
13_C28S_270	C				x					C
13_C9SW_14	C	x		x						C
**13_C9SW_15**	**P**	**x**	**x**	**x**	**x**	**x**	**x**	**x**	**x**	**F2**
13_C9SW_16	C	x								C
13_C26W_3	C			x						C
13_C104W_4	C	x		x						C
**13_C104W_5**	**C**	**x**	**x**	**x**	**x**	**x**	**x**	**x**	**x**	**P**
13_C104W_6	C	x								C
**13_C104W_8**	**C**		**x**	**x**	**x**			**x**	**x**	**C**
**13_C126W_1**	**C**			**x**	**x**				**x**	**F2**
13_C42N_14	C			x						F2
13_C42N_16	C	x		x						C
13_C109N_13	C			x	x					C
**13_C171N_4**	**C**			**x**	**x**			**x**	**x**	**F2**
12_P6SW_1	P	x								P
12_P7S_8	P			x	x					P
12_P8S_76	P				x					P
12_P8S_79	P				x					P
**12_P9S_15**	**P**			**x**	**x**				**x**	**F2**
12_P9S_17	P				x					F2
**12_P25N_23**	**P**			**x**	**x**			**x**	**x**	**F2**
**12_P25N_24**	**P**			**x**	**x**			**x**	**x**	**F2**
13_P4S_205^*^	**P**			**x**	**x**					**F2**
**13_P27S_249**	**P**			**x**	**x**			**x**	**x**	**P**
13_P2SW_38^*^	**P**			**x**	**x**					**F2**
13_P3SW_352	P			x						P
13_P3SW_353	P			x	x				x	P
13_P3SW_354^*^	P				x					P
**13_P26SW_15**	**P**			**x**	**x**					**F2**
13_P56N_7^*^	P				x					P
13_P178N_9^*^	P				x					P
13_P178N_22	P			x						F2

**Notes.**

C *M. capensis* P *M paradoxus* F2 Second generation hybrid

Structure models (i)–(iv) described in the text. Individuals labelled as per Fig. 1. Individuals in bold were used in further validation analyses.

* Non-overlapping confidence intervals.

**Table 4:** Single nucleotide polymorphisms and a 10 bp indel based on the alignment of the flanking region of the microsatellite Mmerhk-3b for *M. capensis*, *M. paradoxus* and *M. merluccius*. The 19 putative hybrids were not heterozygous for any of the positions (accession numbers: KU707035– KU707093).

Species	62 bp	106 bp	120 bp	123–132 bp	156 bp	189 bp
*M. paradoxus*	C	A	T	CTAATTACTA	T	A
*M. capensis*	T	G	C	–	C	G
*M. merluccius*	C	G	C	–	T	G

Assignment tests using the two additional datasets identified different numbers of putative hybrids (Table S3). While dataset 2 (8 loci, null allele frequencies <15%) identified approximately the same individuals as before (Table S3), removing the two loci with fixed differences between species (dataset 3, 6 loci) resulted in a significant increase of the number of admixed individuals identified (Table S3), resulting in a decrease in accuracy, with a higher number of “pure” individuals classified as hybrids (Table S3). However, out of the 108 individuals identified as hybrids, only four exhibit non-overlapping Bayesian credibility intervals. Therefore, the majority of admixed individuals identified reflected the lack of statistical power of the microsatellites.

In the real dataset, only two individuals appeared to exhibit a mito-nuclear discordance: one *M. capensis* sample appeared to have the nuclear genotype of *M. paradoxus* and mtDNA haplotype of *M. capensis*, and another *M. capensis* sampled was identified as *M. paradoxus* based on mtDNA, but had the genotype of *M. capensis* (Table 3).

### Verification of hybrids

**Figure 5:**
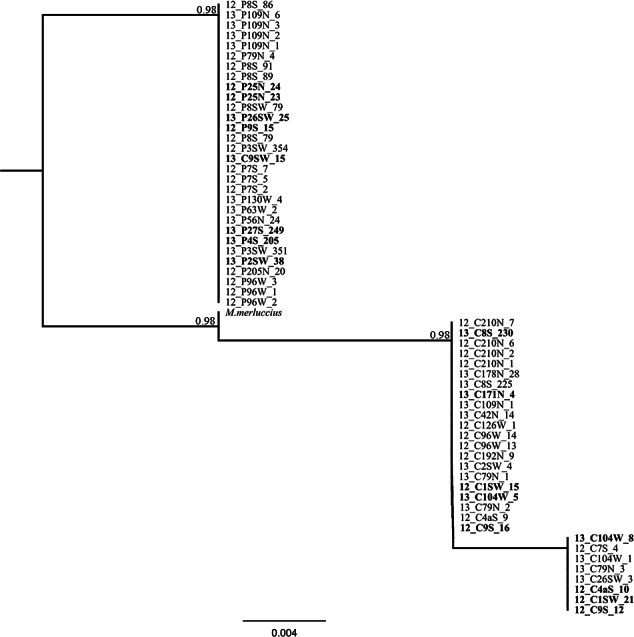
Phylogenetic tree based on locus Mmerhk-3b. Reconstruction of phylogenetic relationships for *M. capensis* and *M. paradoxus*, based on 190 bp of the flanking region of the microsatellite locus Mmerhk-3b. Nodal support indicated above the branches.

Amplification and sequencing of the flanking region of the microsatellite Mmerhk-3b resulted in a fragment of 190 bp for 38 pure individuals (20 *M. paradoxus* and 18 *M. capensis*), with five fixed nucleotide differences and one 10 bp indel observed between the two species (Table 4). The sequence from *M. merluccius* exhibited the same 10 bp deletion than found in *M. capensis*, and had a mix of the fixed positions between *M. capensis* and *M. paradoxus* (Table 4). A total of 19 putative hybrids, identified based on multiple methods and models, were sequenced for the same region (including the two samples with mito-nuclear discordance). No heterozygotic sites were observed for the five fixed positions that differentiate the two hake species, and reconstruction of phylogenetic relationships, using K80 as the model for nucleotide substitution, retrieved two monophyletic clades, one for *M. paradoxus* and one for *M. capensis* (Fig. 5). In particular, individuals that had allele 227 (*M. capensis*) and 241 (*M. paradoxus*) showed no heterozygote sites in the flanking region. The two *M*. *capensis* individuals identified as mito-nuclear discordances grouped either with *M. paradoxus* (13_C9SW_15) or with *M. capensis* (13_C104W_5) for both re-sequenced mtDNA and Mmerhk-3b markers, suggesting that the original discordance was likely the result of a mislabelling error (13_C9SW_15) and microsatellite artefact (13_C104W_5).

## Discussion

*Merluccius paradoxus* and *M. capensis* are co-distributed from the Cunene River Mouth, in northern Namibia, to the Eastern Cape province in South Africa, and are not sister-species (Botha, 1985; Grant & Leslie, 2001; Quinteiro, Vidal & Rey-Mendez, 2000; Von der Heyden, Lipinski & Matthee, 2007a). At the ecological scale, the distributions of adults of the two species are at most predominantly parapatric, with only older *M. capensis* co-existing with younger *M. paradoxus* at the same depth, and fixed differences in allozyme profiles separate the species (Botha, 1985; Grant, Becker & Leslie, 1988; Grant & Leslie, 2001). Recent studies suggest that overlapping of spawning grounds between both species are confined to the West coast of South Africa, and no spawning *M. paradoxus* adults were documented in Namibia (Jansen et al., 2015; Stromme, Lipinski & Kainge, 2015). Despite this, Miralles, Machado-Schiaffino & Garcia-Vazquez (2014) described an extensive north-south latitudinal hybridization gradient, with hake hybrids only found in northern Namibia (where *M. paradoxus* spawning are more than likely absent). Only *M. capensis* individuals had admixed origins, and extensive introgression was reported for *M. paradoxus* caught in northern Namibia (*M. capensis* mtDNA haplotypes and *M. paradoxus* genotypes). Although we included a far greater sample size, with better geographical coverage, we did not observe evidence for extensive hybridization. Not only was there no obvious latitudinal trend in the distribution of putative hybrids, but more importantly, no signs of mito-nuclear discordance were detected after correcting for misidentified individuals.

A careful analysis of the methods employed in the previous hybridization study reveals four major pitfalls. First, the number of markers and individuals employed was limited and all loci were cross-specific. Secondly, the suitability of the microsatellite dataset was not tested to distinguish between the two species and among different hybrid states. Thirdly, although two different Bayesian clustering methods were employed, the authors only used one assignment model to estimate admixture levels (model iv). And finally, the authors did not account for the possibility that either incomplete lineage sorting (Richards & Hobbs, 2015; Van Herwerden et al., 2006) or size homoplasy (Angers & Bernatchez, 1997; Estoup, Jarne & Cornuet, 2002; Grimaldi & CrouauRoy, 1997; Van Oppen et al., 2000) could mimic the effects of interbreeding and result in false hybrids discovery. The lack of consideration to all four of these caveats is not unique to the reported hybridization in *Merluccius*, but is often found throughout the literature (Table S1). Indeed, the majority of studies reviewed used small sample sizes combined with few microsatellite markers, and in these instances very few performed simulations to determine the accuracy of the loci to detect multiple introgression events or accounted for the possibility of incomplete lineage sorting (Richards & Hobbs, 2015; Van Herwerden et al., 2006). Furthermore, the influence of homoplasy in the evolution of microsatellites, although a long known pitfall (Grimaldi & CrouauRoy, 1997), has seldom been explicitly tested.

Previous research demonstrated that the number and type of loci, as well as the number of individuals, has a significant impact on the accuracy of assignment tests, with the use of more than ten loci and over 30 individuals per population recommended to clearly distinguish between “pure” individuals and hybrids (Bohling, Adams & Waits, 2013; Vähä & Primmer, 2006; Väli et al., 2010). However, Miralles, Machado-Schiaffino & Garcia-Vazquez (2014) relied on six cross-specific microsatellite loci, with an average number of alleles per population ranging from 14–21 in *M. paradoxus* and 16–20 in *M. capensis*. Cross-specific loci may decrease the accuracy of the dataset to distinguish between “pure” and hybrid status (Thielsch et al., 2012). To understand how the choice of marker type and number of loci can impact the accuracy of assignments, we used a combination of species-specific (six) and cross-specific (three—the same used in the previous study) loci, and tested three different datasets ranging from nine to six loci (average allelic richness ranging from 5 to 48). Dataset 1 (nine loci) and Dataset 2 (eight loci) did not show differences, even though a locus with a high frequency of null alleles was removed. These results suggest that the assignment methods were not significantly impacted by the presence of null alleles (Carlsson, 2008; Cornuet et al., 1999; Hauser et al., 2006). However, decreasing the number of loci to six (Dataset 3) impacts the accuracy of hybrid detection by increasing the number of admixed individuals (Bohling, Adams & Waits, 2013; Vähä & Primmer, 2006; Väli et al., 2010). It is clear that loci MP318, MP8450 (first used here) and Mmerhk-3b (shared between both studies) are diagnostic markers between these two species and greatly contribute to the accuracy of the assignment tests. These results suggest that by using only six markers, with only one being suitable for species differentiation, might have contributed to the overestimation of the number of admixed individuals in Miralles, Machado-Schiaffino & Garcia-Vazquez (2014).

Furthermore, several studies have highlighted the need to conduct *a priori* simulations when using Bayesian approaches for assigning individuals to populations (Bohling, Adams & Waits, 2013; Hoban, 2014; Vähä & Primmer, 2006). The majority of available clustering methods rely on determining ancestry coefficients, making it necessary to first establish a baseline, as it is generally difficult to relate them to true genetic ancestry (Bohling, Adams & Waits, 2013; Vähä & Primmer, 2006). Simulation studies are, thus, essential for assessing type I and type II errors, and for minimizing the occurrence of false positives. Our simulation results show that detection of hybrids between *M. paradoxus* and *M. capensis* clearly depends on the number of markers used, and the model and approaches. As pointed out by Väli et al. (2010), these findings imply that in the case of a limited number of microsatellite loci available, multiple methods should be employed to achieve greater accuracy.

Assessment of the performance of the four admixture models available in STRUCTURE also retrieved different results. As expected, No Admixture Models (i and ii) identified a smaller number of individuals with mixed origins, when compared with the Admixture Models (iii and iv). The statistical framework of this method thus profoundly influences its outcomes (Bohling, Adams & Waits, 2013; Hoban, 2014; Vähä & Primmer, 2006). In particular, model iv (Admixture, Correlated Allelic Frequencies) appears to overestimate the number of hybrids available, by misclassifying “pure” individuals as putative hybrids. Although there is a biological reasoning behind using such a model in studies of hybridization between populations of the same species, or between recently diverged species (Pritchard, Stephens & Donnelly, 2000), our results show that this is not the most suitable approach when species have been isolated for longer periods, as the assumptions of interbreeding and related allelic frequencies are likely not to be met.

### Interbreeding, ancestral polymorphism or homoplasy?

The microsatellite dataset used in this study revealed the presence of multiple individuals with admixed ancestry, 19 of which were consistently identified as *F*_2_ hybrids by different models and approaches. The majority of hybridization studies would thus accept these 19 individuals as hybrids, with an interbreeding rate of 1.58% between *M. paradoxus* and *M. capensis*. However, there is a distinct caveat in using microsatellites when making such assumptions in species that have evolutionary divergent histories, as microsatellite loci do not evolve in the same manner as other genomic sequences (Calabrese & Sainudiin, 2005; Ellegren, 2004). For example, a study between two sister-species of coral trouts (*Plectropomus*) revealed a complex pattern of hybridization, from ancestral introgression, and incomplete lineage sorting at one microsatellite locus, to complete reproductive isolation (Van Herwerden et al., 2006). In this case, the two species had been isolated for only 500,000 years. In another case, cichlid species isolated for 700,000 years exhibited evidence of homoplasic mutations in one locus (Van Oppen et al., 2000). These cases illustrate the need to assess the accuracy of microsatellites for distinguishing between interbreeding, retention of ancestral polymorphisms through incomplete lineage sorting and homoplasy.

The sequencing of the most divergent microsatellite loci (Mmerhk-3b) revealed five fixed nucleotide substitutions (all transitions) and one 10 bp insertion in *M. paradoxus*. Reconstruction of phylogenetic relationships clearly distinguished between the two hake species, and all putative hybrids were assigned to either clade, with no heterozygotes observed. These findings support a hypothesis of no hybridization between *M. paradoxus* and *M. capensis*, corroborating earlier allozyme studies (Grant & Leslie, 2001). We then used the available sequence of Mmerhk-3b of *M. merluccius*, the sister-species of *M. capensis*, in order to understand if retention of ancestral polymorphism could explain the presence of same-size alleles in the two species. *Merluccius merluccius* grouped with *M. capensis*, shared nucleotide changes with both species, but did not have the 10 bp insertion observed in *M. paradoxus*. Rare genomic changes such as indels are considered good indicators of phylogenetic relationships, as their presence is highly conserved across groups of animals (Matthee et al., 2007). The absence of the 10 bp insertion in both *M. merluccius* and *M. capensis* suggests that these species share a common ancestor (as observed by their close phylogenetic relationship). Therefore, it is not likely that the presence of same-size alleles in *M. paradoxus* and *M. capensis* results from the retention of ancestral polymorphisms in this case since they share no homologous alleles at this locus (Campo et al., 2007; Grant & Leslie, 2001; Quinteiro, Vidal & Rey-Mendez, 2000). It is more likely that complete lineage sorting has occurred, at least for this marker. Furthermore, as the changes in the flanking region were observed in both putative hybrids and pure individuals, it is not likely that the alleles were obtained via heterospecific crossing. These findings point to size homoplasy as the most probable mechanism behind the identification of putative hybrids between these species.

Homoplasy in microsatellites is generally expressed as changes in the repeat unit (point mutations, compound microsatellites) or mutations in the flanking region (Angers & Bernatchez, 1997; Estoup, Jarne & Cornuet, 2002; Grimaldi & CrouauRoy, 1997; Van Oppen et al., 2000), as observed in Mmerhk-3b. Nevertheless, the use of only one marker under selection (Hoareau et al., 2015), to make inferences about genome-wide homoplasy has in itself several drawbacks. First, recombination and unequal gene conversion can, by chance, mimic the effects of homoplasy and thus influence the conclusions (Balaresque et al., 2014; Ziegler et al., 2009). However, the mutation rate within the repeat unit is generally higher than the recombination rate between the core sequence and the flanking region (10^−6^ for sequences 100 bp long—Hilliker, Clark & Chovnick, 1991), making it unlikely for recombination or gene conversion to be responsible for the observed differences (Estoup, Jarne & Cornuet, 2002). Second, the ability to detect multiple generation hybrids using nuclear sequences is somewhat limited, as the probability of identifying them decreases with an increase in repeated events (F1–100%; F2–33%; backcrosses–16.67%). For that reason, we specifically targeted individuals that were either heterozygotes or homozygotes for alleles of the other species. If these admixed individuals resulted from heterospecific crossings then we would expect the flanking region to either show heterozygotes for the fixed positions (as they had one allele from each species), or the diagnostic SNPs from the other species (for homozygotes). That was not the case, which further supports the hypothesis of homoplasy instead of hybridization between the Cape hakes. Finally, Mmerhk-3b exhibited significant deviations to neutrality, and thus selection against hybrids might have influenced our findings (by removing heterozygotes from the gene pool). As mentioned above, we selected individuals exhibiting the alleles common to the other species, and so, would still expect to retrieve heterozygotes for the fixed positions. Therefore, it is likely that the observed changes in Mmerhk-3b indeed reflect homoplasy and not heterospecific crossings between the two Cape hake species.

Size homoplasy has long been known to affect microsatellite loci, as it is deeply linked to the most common mutational model for these markers (SSM: Single Stepwise Model) (Angers & Bernatchez, 1997; Estoup, Jarne & Cornuet, 2002; Grimaldi & CrouauRoy, 1997; Van Oppen et al., 2000). In addition, evolutionary factors such as mutation rate, effective population size and time since divergence can also influence homoplasic loci (reviewed in Estoup, Jarne & Cornuet, 2002). Empirical studies suggest that for an average mutation rate of 5 × 10^−4^ all modelled alleles are homoplasic after 6,000 generations, regardless of effective population sizes and mutational models (Estoup, Jarne & Cornuet, 2002). With an average generation time of 3.5 years (Botha, 1985) 6,000 generations would correspond to 21,000 years for the Cape Hakes. As the species have been isolated for 3–4 Ma, it is thus likely that similar allelic forms result from widespread homoplasy, and not heterospecific crossings (Estoup, Jarne & Cornuet, 2002).

Nevertheless, a large number of studies in population genetics in the marine environment continues to rely on these markers to describe hybridization and often the analytical methods to make inferences are not fully explored (Table S1). This is particularly concerning when the species are commercially exploited since accurate evolutionary histories are required for adequate management. In conclusion, our study provides empirical data showing that homoplasy has likely mimicked the effects of heterospecific crossings, and resulted in false positive signals of hybridization between Cape hakes. This has important implications for the management of these two co-distributed species, as hybridization plays no obvious part in the genetic structuring of these species.

## Supplemental Information

10.7717/peerj.1827/supp-1 Supplemental Information 1 Microsatellite database Microsatellite database for *M. capensis* and *M. paradoxus*

10.7717/peerj.1827/supp-2 Figure S1 Depicting the distribution of allelic frequencies for *M. capensis* and *M. paradoxus* Distribution of allelic frequencies of *M. capensis* (white) and *M. paradoxus* (grey) by microsatellite locus.

10.7717/peerj.1827/supp-3 Figure S2 Showing the genetic identification of samples Identification of *M. capensis* (white) and *M. paradoxus (grey)* based on the CR of mtDNA (A) and genotype frequencies as obtained in STRUCTURE for nine microsatellite loci (B).

10.7717/peerj.1827/supp-4 Table S1 Annex 1 Papers reviewed on the subject of hybridization between marine species, with information on species, genetic relationships and divergence levels between hybridizing species, genetic marker used and clustering methods employed to describe hybrids.

10.7717/peerj.1827/supp-5 Table S2 Annex 2 *Merluccius capensis* and *M. paradoxus* individuals identified as hybrids based on nine microsatellite loci (I), eight microsatellite loci (II) and six microsatellite loci (III): C—*M. capensis*, P—*M paradoxus*, F2—second generation hybrid, BcP—backcross with *M. paradoxus*. Individuals labelled as per Fig. 1.

10.7717/peerj.1827/supp-6 Table S3 Annex 3 Supporting references for Table S1.

## Additional Information and Declarations

### DNA Deposition

The following information was supplied regarding the deposition of DNA sequences: MtDNA sequences accession numbers: KU705901– KU707034
Mmerhk-3b sequences accession numbers: KU707035– KU707093.

### Data Availability

The following information was supplied regarding data availability: DRYAD: 10.5061/dryad.8673j.
