# Supplementary figures and images for: When homoplasy mimics hybridization: a case study of Cape hakes (Merluccius capensis and M. paradoxus)
Source: PeerJ. 2016 Mar 28;4:e1827. doi: 10.7717/peerj.1827 (PMC4824878; doi:10.7717/peerj.1827)

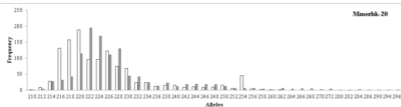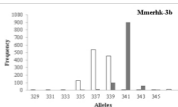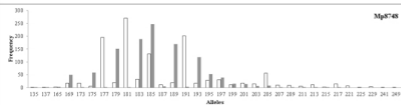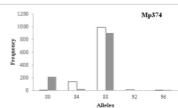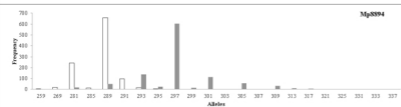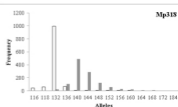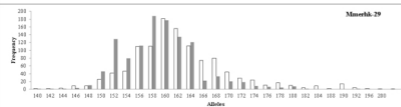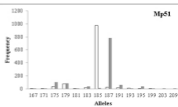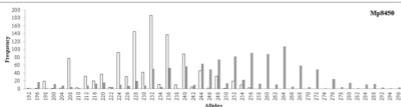

Supplement: Figure S1 — Distribution of allelic frequencies of M. capensis (white) and M. paradoxus (grey) by microsatellite locus. [file peerj-04-1827-s002.pdf]

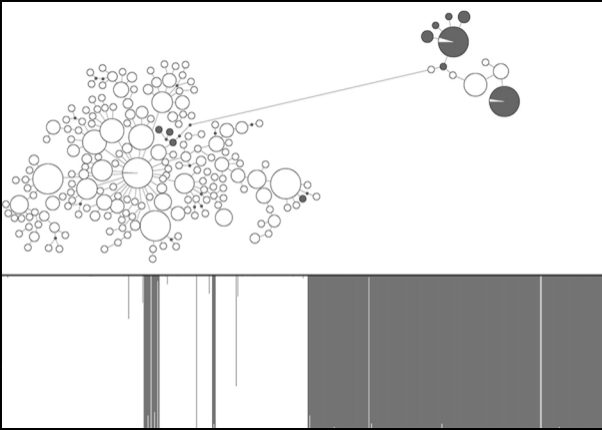

Supplement: Figure S2 — Identification of M. capensis (white) and M. paradoxus (grey) based on the CR of mtDNA (A) and genotype frequencies as obtained in STRUCTURE for nine microsatellite loci (B). [file peerj-04-1827-s003.pdf]
