# Supplementary material for: When homoplasy mimics hybridization: a case study of Cape hakes (Merluccius capensis and M. paradoxus)
Source: PeerJ. 2016 Mar 28;4:e1827. doi: 10.7717/peerj.1827 (PMC4824878; doi:10.7717/peerj.1827)
Supplement: Table S1 — Papers reviewed on the subject of hybridization between marine species, with information on species, genetic relationships and divergence levels between hybridizing species, genetic marker used and clustering methods employed to describe hybrids. [file peerj-04-1827-s004.docx]

Table S1: Papers reviewed on the subject of hybridization between marine species, with information on species, genetic relationships and divergence levels between hybridizing species, genetic marker used and clustering methods employed to describe hybrids

| **Reference** | **Species** | **Sister-species** | **mtDNA divergence** | **Occurrence** | **Marker** | **Clustering methods** | | |
| --- | --- | --- | --- | --- | --- | --- | --- | --- |
|  |  |  |  |  |  | **HybridLab** | **NewHybrids** | **Structure** |
| McMillan *et al* (1999) | *C. punctatofasciatus / C. pelewensis* | no | 0.007 | sympatry | mtDNA | 0 | 0 | 0 |
| Roques *et al* (2001) | *S. fasciatus / S. mentella* | yes | 0.03 | allopatry - hybrid zone | microsats | 0 | 0 | 0 |
| McCartney *et al* (2003) | 6 morphospecies | yes | 0.006 | sympatry | microsats | 0 | 0 | 0 |
| Nielsen *et al* (2003) | *G. morhua* | intraspecific | <0.01 | allopatry - hybrid zone | microsats | 1 | 0 | 1 |
| Addison and Hart (2005) | *S. droebachinensis / S. pallidus* | no | 0.039 | sympatry | microsats | 0 | 0 | 0 |
| Yaakub *et al* (2006) | *T. jansenii / T. quinquevittatum* | no | 0.02 | sympatry | S7 and other nDNA | 0 | 0 | 0 |
| van Herwerden *et al* (2006) | *P. leopardus / P. maculatus* | yes | 0.01 | allopatry - hybrid zone | Microsats | 0 | 0 | 0 |
| Yaakub *et al* (2007) | *H. garnoti / H. bivittatus* | yes | 0.055 | sympatry | S7 | 0 | 0 | 0 |
| Marie *et al* (2007) | *A. leucosternon / A. nigricans* | yes | 0.01 | sympatry | S7 | 0 | 0 | 0 |
| Kuriiwa *et al* (2007) | *S. guttatus / S. lineatus; S. virgatus/S. doliatus* | yes | 0.01 | sympatry | ITS | 0 | 0 | 0 |
|  | *S. corallinus/S. puellus* | no | 0.1 | sympatry | ITS | 0 | 0 | 0 |
|  | *S. corallinus A/S. corallinus B* | yes | 0.064 | allopatry - hybrid zone | ITS | 0 | 0 | 0 |
| Roberts *et al* (2009) | *A. australis / A. butcheri* | no | 0.043 | sympatry | microsats | 0 | 1 | 1 |
| Machado-Schiaffino *et al* (2010) | *M. albidus / M. bilinearis* | no | 0.077 | sympatry | microsats | 0 | 0 | 1 |
| Montanari *et al* (2012) | *C. trifasciatus / C. lunulatus* | yes | 0.05 | allopatry - hybrid zone | microsats | 0 | 0 | 0 |
| Ouanes *et al* (2011) | *S. aegyptica / S. senegalensis* | yes | 0.087 | allopatry - hybrid zone | EPIC-PCR | 0 | 0 | 1 |
| Burford *et al* (2011) | *S. mystinus* | intraspecific | 0.014 | allopatry - hybrid zone | microsats | 0 | 1 | 1 |
| Mullen *et al* (2012) | *S. diencaeus / S. adustus* | yes | 0.03 | sympatry | S7 | 0 | 0 | 0 |
| Vilaca *et al* (2012) | *C. caretta / E. imbricata / L. olivacea /C. mydas* | no | 0.045 | sympatry | microsats | 1 | 1 | 1 |
| Attard *et al* (2012) | *B. m. brevicauda / B. m. intermedia* | yes | 0.12, Microsats) | sympatry | microsats | 1 | 1 | 1 |
| von der Heyden and Connell (2012) | *C. anglicus/C.puniceus* | no | 0.11 | sympatry | nuclear genes | 0 | 0 | 0 |
| Miralles *et al* (2013) | *G. melas / G. macrorhynchus* | yes | 0.019 | sympatry | microsats | 0 | 1 | 1 |
| Muto *et al* (2013) | *S. vulpes / S. zonatus* | yes | 0.015 | sympatry | AFLPs | 0 | 0 | 1 |
| Crego-Prieto *et al* (2012) | *L. whiffiagonis / L. boscii* | yes | 0.086 | sympatry | microsats | 0 | 1 | 1 |
| Miralles *et al* (2014) | *M. capensis / M. paradoxus* | no | 0.077 | sympatry | microsats | 0 | 1 | 1 |
| Amaral *et al* (2014) | *S. clymene / S. longirostris / S. coeruleoalba* | yes | 0.03 | sympatry | nDNA | 0 | 0 | 0 |
| Potts *et al* (2014) | *A. coronus / A. inodorus* | no | 0.037 | allopatry - hybrid zone | microsats | 1 | 1 | 1 |
| Mirimin *et al* (2014) | *A. inodorus / A. japonicus* | no | 0.049 | sympatry | microsats | 0 | 1 | 1 |
| Sanders *et al* (2014) | *A. fuscus / A. laevis* | yes | 0.004 | sympatry | microsats | 1 | 0 | 1 |
| Montanari *et al* (2014) | *C. guttatissimus / C. puntatofasciatus* | yes | 0.01 | allopatry - hybrid zone | microsats | 0 | 1 | 1 |
| Coleman *et al* (2014) | *A. abdominalis / A. vaigiensis* | no | 0.04 | sympatry | S7 and other nDNA | 1 | 1 | 1 |
| Bradbury *et al* (2014) | *G. morhua* forms | intraspecific | <0.01 | allopatry - hybrid zone | SNPs | 1 | 1 | 1 |
