# Supplementary material for: When homoplasy mimics hybridization: a case study of Cape hakes (Merluccius capensis and M. paradoxus)
Source: PeerJ. 2016 Mar 28;4:e1827. doi: 10.7717/peerj.1827 (PMC4824878; doi:10.7717/peerj.1827)
Supplement: Table S2 — Merluccius capensis and M. paradoxus individuals identified as hybrids based on nine microsatellite loci (I), eight microsatellite loci (II) and six microsatellite loci (III): C—M. capensis, P—M paradoxus, F2—second generation hybrid, BcP—backcross with M. paradoxus. Individuals labelled as per Fig. 1. [file peerj-04-1827-s005.docx]

Table S2: *Merluccius capensis* and *M. paradoxus* individuals identified as hybrids based on nine microsatellite loci (I), eight microsatellite loci (II) and six microsatellite loci (III): C – *M. capensis*, P – *M paradoxus*, F2 – second generation hybrid, BcP – backcross with *M. paradoxus*. Individuals labelled as per Figure 1.

|  | Structure | | | | |  | NewHybrids | | |
| --- | --- | --- | --- | --- | --- | --- | --- | --- | --- |
| Individual | I | | II | | III | |  |  |  |
|  | q=0.1 | q=0.2 | q=0.1 | q=0.2 | q=0.1 | q=0.2 | I | II | III |
| 12_C4aS_1 |  |  | x |  |  |  | C |  |  |
| 12_C4aS_4 | x |  | x |  |  |  | F2 | F2 |  |
| 12_C4aS_7 |  |  |  |  | x |  | C |  |  |
| 12_C4aS_9 |  |  | x |  |  |  | C |  |  |
| 12_C4aS_10 | x | x | x | x | x | x | F2 | F2 |  |
| 12_C2S_6 |  |  |  |  | x |  | C |  |  |
| 12_C9S_12 | x | x | x | x |  |  | F2 | F2 | P |
| 12_C9S_16 | x | x | x | x |  |  | F2 | F2 |  |
| 12_C1SW_13 | x |  |  |  |  |  | C |  |  |
| 12_C1SW_15 | x | x | x |  |  |  | F2 | F2 | F2 |
| 12_C1SW_21 | x | x | x |  |  |  | F2 |  |  |
| 12_C1SW_30 |  |  |  |  |  |  | C |  | F2 |
| 12_C11SW_19 |  |  |  |  |  | x | F2 |  |  |
| 12_C102W_3 | x |  | x |  | x | x | C |  | F2 |
| 12_C11SW_19 |  |  |  |  | x |  | C |  | F2 |
| 12_C94W_6 |  |  |  |  | x |  | C |  |  |
| 12_C96W_15 |  |  |  |  | x | x | C |  |  |
| 12_C102W_6 |  |  |  |  | x |  | C |  |  |
| 12_C102W_13 |  |  | x |  |  |  | C |  |  |
| 12_C4N_1 |  |  |  |  |  |  | C |  | P |
| 12_C192N_8 |  |  |  |  | x | x | C |  |  |
| 12_C195bN_2 |  |  |  |  | x |  | C |  |  |
| 12_C196N_19 |  |  |  |  | x |  | C |  |  |
| 12_C1SW_28 |  |  |  |  | x |  | C |  |  |
| 12_C210N_1 |  |  |  |  |  |  | C |  | F2 |
| 12_C210N_24 |  |  |  |  | x |  | C |  |  |
| 12_C210N_4 |  |  |  |  | x | x | C |  | C/P |
| 12_C89N_13 |  |  |  |  | x |  | C |  |  |
| 12_C89N_17 |  |  |  |  | x | x | C |  |  |
| 13_C1S_271 |  |  |  |  | x |  | C |  |  |
| 13_C1S_277 |  |  | x |  | x | x | C |  | F2 |
| 13_C2S_3 |  |  |  |  | x |  | C |  |  |
| 13_C8S_230 | x | x | x | x |  |  | F2 | F2 |  |
| 13_C28S_253 |  |  |  |  | x |  | C |  |  |
| 13_C28S_254 |  |  |  |  | x | x | C |  | P |
| 13_C28S_255 |  |  |  |  | x | x | C |  | F2 |
| 13_C28S_266 |  |  |  |  |  |  | C |  | F2 |
| 13_C28S_270 |  |  |  |  |  |  | C |  | F2 |
| 13_C1SW_18 |  |  |  |  | x | x | C |  |  |
| 13_C2SW_3 |  |  |  |  | x | x | C |  | C/F2 |
| 13_C9SW_14 | x |  |  |  |  |  | C |  |  |
| 13_C9SW_15 | x | x |  |  |  |  | F2 | F2 | P |
| 13_C9SW_16 |  |  |  |  |  |  | C |  |  |
| 13_C10SW_14 |  |  |  |  | x | x | C |  |  |
| 13_C10SW_3 |  |  |  |  | x | x | C |  | F2 |
| 13_C10SW_5 |  |  |  |  | x |  | C |  |  |
| 13_C126SW_19 |  |  |  |  | x |  | C |  |  |
| 13_C26W_3 | x |  | x |  |  |  | C |  |  |
| 13_C76W_5 |  |  |  |  | x | x | C |  |  |
| 13_C79W_17 |  |  |  |  | x | x | C |  | C/F2 |
| 13_C104W_3 |  |  |  |  | x |  | C |  |  |
| 13_C104W_4 | x |  |  |  | x |  | C |  |  |
| 13_C104W_5 | x | x | x | x | x | x | P | P | P |
| 13_C104W_6 |  |  |  |  | x | x | C |  | F2 |
| 13_C104W_8 | x | x | x |  | x | x | C |  | C/P |
| 13_C119W_8 |  |  |  |  | x | x | C |  |  |
| 13_C120W_14 |  |  |  |  | x | x | C |  |  |
| 13_C120W_17 |  |  | x |  | x | x | C |  | F2 |
| 13_C126W_1 | x |  | x | x | x |  | F2 | F2 | F2 |
| 13_C42N_14 | x |  | x |  |  |  | F2 | F2 |  |
| 13_C42N_16 | x |  | x |  |  |  | C |  |  |
| 13_C56N_22 |  |  |  |  | x | x | C |  | P |
| 13_C109N_6 |  |  |  |  | x |  | C |  |  |
| 13_C109N_13 | x |  | x |  | x |  | C |  | P |
| 13_C109N_17 |  |  |  |  | x | x | C |  |  |
| 13_C109N_29 |  |  |  |  |  |  | C |  | P |
| 13_C171N_4 | x | x | x | x |  |  | F2 | F2 | P |
| 13_C171N_10 |  |  |  |  | x |  | C |  |  |
| 13_C171N_12 |  |  |  |  | x |  | C |  | F2 |
| 13_C171N_6 |  |  |  |  | x |  | C |  |  |
| 13_C178N_15 |  |  |  |  | x |  | C |  |  |
| 13_C178N_18 |  |  |  |  | x | x | C |  |  |
| 12_P16SW_48 |  |  | x |  |  |  | C |  |  |
| 12_P6SW_1 |  |  |  |  |  |  | P |  |  |
| 12_P6SW_4 |  |  | x |  |  |  | P |  |  |
| 12_P114W_10 |  |  |  |  | x |  | P |  | C |
| 12_P15SW_10 |  |  |  |  | x | x | P |  |  |
| 12_P16SW_19 |  |  |  |  | x |  | P |  |  |
| 12_P16SW_48 |  |  |  |  | x | x | P |  |  |
| 12_P25aSW_1 |  |  |  |  | x |  | P |  | P/F2 |
| 12_P25aSW_6 |  |  |  |  |  |  | P |  | F2 |
| 12_P25aSW_7 |  |  |  |  | x | x | P |  | F2 |
| 12_P6SW_11 |  |  |  |  | x |  | P |  | F2 |
| 12_P6SW_2 |  |  |  |  | x | x | P |  |  |
| 12_P6SW_4 |  |  |  |  | x | x | P |  |  |
| 12_P6SW_9 |  |  |  |  | x |  | P |  |  |
| 12_P16SW_483 |  |  |  |  |  |  | P |  | F2 |
| 12_P7S_8 | x |  |  |  |  |  | P |  |  |
| 12_P8S_73 |  |  |  |  | x | x | P |  | C |
| 12_P8S_76 |  |  |  |  |  |  | P | F2 | C |
| 12_P8S_79 |  |  | x | x | x |  | P | F2 | F2 |
| 12_P8S_86 |  |  |  |  | x | x | P |  | C |
| 12_P9S_15 | x |  |  |  |  |  | F2 |  |  |
| 12_P9S_17 |  |  |  |  |  |  | F2 |  |  |
| 12_P25N_1 |  |  |  |  |  |  | P |  | BcP |
| 12_P25N_12 |  |  |  |  | x |  | P |  |  |
| 12_P25N_15 |  |  |  |  | x | x | P |  | P/F2 |
| 12_P25N_22 |  |  |  |  | x |  | P |  |  |
| 12_P25N_23 | x | x | x | x |  |  | F2 | F2 |  |
| 12_P25N_24 | x | x | x | x |  |  | F2 | F2 |  |
| 12_P79N_4 |  |  | x |  | x | x | P |  | F2 |
| 12_P79N_12 |  |  |  |  | x |  | P |  |  |
| 12_P79N_17 |  |  |  |  | x |  | P |  |  |
| 12_P79N_9 |  |  |  |  | x | x | P |  | F2 |
| 12_P191N_15 |  |  |  |  | x | x | P |  | C |
| 12_P191N_17 |  |  |  |  | x |  | P |  |  |
| 12_P205N_6 |  |  |  |  | x | x | P |  | F2 |
| 12_P205N_24 |  |  |  |  |  |  | P |  | F2 |
| 12_P211N_1 |  |  |  |  | x | x | P |  | F2 |
| 12_P211N_8 |  |  |  |  | x |  | P |  | C |
| 13_P4S_205 | x |  | x |  |  |  | F2 | F2 | F2 |
| 13_P27S_249 | x | x | x | x |  |  | F2 |  | C |
| 13_P22S_304 |  |  |  |  | x |  | P |  |  |
| 13_P27S_239 |  |  |  |  | x | x | P |  | F2 |
| 13_P1SW_4 |  |  |  |  | x | x | P |  |  |
| 13_P3SW_4 |  |  |  |  |  |  | P |  | C |
| 13_P12SW_17 |  |  |  |  | x |  | P |  |  |
| 13_P12SW_3 |  |  |  |  | x |  | P |  |  |
| 13_P29SW_18 |  |  |  |  | x |  | P |  |  |
| 13_P29SW_7 |  |  |  |  | x | x | P |  |  |
| 13_P29SW_8 |  |  |  |  | x | x | P |  |  |
| 13_P2SW_37 |  |  |  |  | x | x | P |  |  |
| 13_P2SW_38 | x |  |  |  | x | x | F2 |  | F2 |
| 13_P3SW_352 | x |  |  |  | x | x | P |  | F2 |
| 13_P3SW_353 | x |  | x | x | x |  | P | F2 |  |
| 13_P3SW_354 |  |  |  |  | x | x | P |  | F2 |
| 13_P3SW_366 |  |  |  |  | x |  | P |  |  |
| 13_P26SW_15 | x |  | x | x | x | x | F2 | F2 | F2 |
| 13_P26SW_19 |  |  |  |  |  |  | P |  | F2 |
| 13_P29SW_7 |  |  |  |  |  |  | P |  | P/F2 |
| 13_P29SW_8 |  |  | x |  |  |  | P |  | F2 |
| 13_P85SW_9 |  |  |  |  | x | x | P |  | C |
| 13_P63W_12 |  |  |  |  | x |  | P |  |  |
| 13_P63W_7 |  |  |  |  | x | x | P |  | F2 |
| 13_P93W_3 |  |  |  |  | x |  | P |  |  |
| 13_P93W_4 |  |  |  |  | x |  | P |  | F2 |
| 13_P100W_11 |  |  |  |  | x | x | P |  |  |
| 13_P100W_14 |  |  |  |  | x |  | P |  |  |
| 13_P100W_19 |  |  |  |  | x | x | P |  | P/F2 |
| 13_P130W_2 |  |  |  |  |  |  | P |  | F2 |
| 13_P42N_10 |  |  |  |  | x |  | P |  |  |
| 13_P56N_10 |  |  |  |  | x |  | P |  |  |
| 13_P56N_11 |  |  |  |  | x | x | P |  |  |
| 13_P56N_13 |  |  |  |  | x | x | P |  |  |
| 13_P56N_20 |  |  |  |  | x | x | P |  |  |
| 13_P56N_24 |  |  |  |  | x | x | P |  |  |
| 13_P92N_13 |  |  |  |  | x | x | P |  |  |
| 13_P92N_6 |  |  |  |  | x | x | P |  | F2 |
| 13_P56N_7 |  |  | x |  | x | x | P |  | F2 |
| 13_P56N_11 |  |  |  |  |  |  | P |  | C/P |
| 13_P56N_13 |  |  |  |  |  |  | P |  | F2 |
| 13_P178N_9 |  |  |  |  | x | x | P | F2 | F2 |
| 13_P178N_21 |  |  |  |  | x |  | P |  | F2 |
| 13_P178N_22 | x |  |  |  |  |  | F2 |  |  |
| **TOTAL** | **31** | **13** | **33** | **13** | **108** | **59** | **22** | **19** | **68** |
