# Supplementary material for: When homoplasy mimics hybridization: a case study of Cape hakes (Merluccius capensis and M. paradoxus)
Source: PeerJ. 2016 Mar 28;4:e1827. doi: 10.7717/peerj.1827 (PMC4824878; doi:10.7717/peerj.1827)
Supplement: Table S3 — Supporting references for Table S1. [file peerj-04-1827-s006.docx]

**References**

Addison JA, Hart MW (2005). Colonization, dispersal, and hybridization influence phylogeography of North Atlantic sea urchins (*Strongylocentrotus droebachiensis*). *Evolution* **59**(3)**:** 532-543.

Amaral AR, Lovewell G, Coelho MM, Amato G, Rosenbaum HC (2014). Hybrid speciation in a marine mammal: the Clymene dolphin (S*tenella clymene*). *Plos One* **9**(1).

Attard CRM, Beheregaray LB, Jenner KCS, Gill PC, Jenner M-N, Morrice MG *et al* (2012). Hybridization of southern hemisphere blue whale subspecies and a sympatric area off Antarctica: impacts of whaling or climate change? *Molecular Ecology* **21**(23)**:** 5715-5727.

Bradbury IR, Bowman S, Borza T, Snelgrove PVR, Hutchings JA, Berg PR *et al* (2014). Long distance linkage disequilibrium and limited hybridization suggest cryptic speciation in Atlantic cod. *Plos One* **9**(9).

Burford MO, Bernardi G, Carr MH (2011). Analysis of individual year-classes of a marine fish reveals little evidence of first-generation hybrids between cryptic species in sympatric regions. *Marine Biology* **158**(8)**:** 1815-1827.

Coleman RR, Gaither MR, Kimokeo B, Stanton FG, Bowen BW, Toonen RJ (2014). Large-scale introduction of the Indo-Pacific damselfish *Abudefduf vaigiensis* into Hawai'i promotes genetic swamping of the endemic congener *A. abdominalis*. *Molecular Ecology* **23**(22)**:** 5552-5565.

Crego-Prieto V, Martinez JL, Roca A, Garcia-Vazquez E (2012). Interspecific hybridization increased in congeneric flatfishes after the Prestige oil spill. *Plos One* **7**(4).

Kuriiwa K, Hanzawa N, Yoshino T, Kimura S, Nishida M (2007). Phylogenetic relationships and natural hybridization in rabbitfishes (Teleostei : Siganidae) inferred from mitochondrial and nuclear DNA analyses. *Molecular Phylogenetics and Evolution* **45**(1)**:** 69-80.

Machado-Schiaffino G, Juanes F, Garcia-Vazquez E (2010). Introgressive hybridization in North American hakes after secondary contact. *Molecular Phylogenetics and Evolution* **55**(2)**:** 552-558.

Marie AD, Van Herwerden L, Choat JH, Hobbs JPA (2007). Hybridization of reef fishes at the Indo-Pacific biogeographic barrier: a case study. *Coral Reefs* **26**(4)**:** 841-850.

McCartney MA, Acevedo J, Heredia C, Rico C, Quenoville B, Bermingham E *et al* (2003). Genetic mosaic in a marine species flock. *Molecular Ecology* **12**(11)**:** 2963-2973.

McMillan WO, Weigt LA, Palumbi SR (1999). Color pattern evolution, Assortative mating, and genetic differentiation in brightly colored butterflyfishes (Chaetodontidae). *Evolution* **53**(1)**:** 247-260.

Miralles L, Lens S, Rodriguez-Folgar A, Carrillo M, Martin V, Mikkelsen B *et al* (2013). Interspecific introgression in cetaceans: DNA markers reveal post-F1 status of a pilot whale. *Plos One* **8**(8).

Miralles L, Machado-Schiaffino G, Garcia-Vazquez E (2014). Genetic markers reveal a gradient of hybridization between cape hakes (*Merluccius capensis* and *Merluccius paradoxus*) in their sympatric geographic distribution. *Journal of Sea Research* **86:** 69-75.

Mirimin L, Kerwath SE, Macey BM, Bester-van der Merwe AE, Lamberth SJ, Bloomer P *et al* (2014). Identification of naturally occurring hybrids between two overexploited sciaenid species along the South African coast. *Molecular Phylogenetics and Evolution* **76:** 30-33.

Montanari SR, Hobbs J-PA, Pratchett MS, Bay LK, Van Herwerden L (2014). Does genetic distance between parental species influence outcomes of hybridization among coral reef butterflyfishes? *Molecular Ecology* **23**(11)**:** 2757-2770.

Montanari SR, Van Herwerden L, Pratchett MS, Hobbs J-PA, Fugedi A (2012). Reef fish hybridization: lessons learnt from butterflyfishes (Genus *Chaetodon*). *Ecology and Evolution* **2**(2)**:** 310-328.

Mullen SP, Little K, Draud M, Brozek J, Itzkowitz M (2012). Hybridization among Caribbean damselfish species correlates with habitat degradation. *Journal of Experimental Marine Biology and Ecology* **416:** 221-229.

Muto N, Kai Y, Noda T, Nakabo T (2013). Extensive hybridization and associated geographic trends between two rockfishes *Sebastes vulpes* and *S. zonatus* (Teleostei: Scorpaeniformes: Sebastidae). *Journal of Evolutionary Biology* **26**(8)**:** 1750-1762.

Nielsen EE, Hansen MM, Ruzzante DE, Meldrup D, Gronkjaer P (2003). Evidence of a hybrid-zone in Atlantic cod (*Gadus morhua*) in the Baltic and the Danish Belt Sea revealed by individual admixture analysis. *Molecular Ecology* **12**(6)**:** 1497-1508.

Ouanes K, Bahri-Sfar L, Ben Hassine OK, Bonhomme F (2011). Expanding hybrid zone between *Solea aegyptiaca* and *Solea senegalensis:* genetic evidence over two decades. *Molecular Ecology* **20**(8)**:** 1717-1728.

Potts WM, Henriques R, Santos CV, Munnik K, Ansorge I, Dufois F *et al* (2014). Ocean warming, a rapid distributional shift, and the hybridization of a coastal fish species. *Global Change Biology* **20**(9)**:** 2765-2777.

Roberts DG, Gray CA, West RJ, Ayre DJ (2009). Evolutionary impacts of hybridization and interspecific gene flow on an obligately estuarine fish. *Journal of Evolutionary Biology* **22**(1)**:** 27-35.

Roques S, Sevigny JM, Bernatchez L (2001). Evidence for broadscale introgressive hybridization between two redfish (Genus *Sebastes*) in the North-west Atlantic: a rare marine example. *Molecular Ecology* **10**(1)**:** 149-165.

Sanders KL, Rasmussen AR, Guinea ML (2014). High rates of hybridisation reveal fragile reproductive barriers between endangered Australian sea snakes. *Biological Conservation* **171:** 200-208.

van Herwerden L, Choat JH, Dudgeon CL, Carlos G, Newman SJ, Frisch A *et al* (2006). Contrasting patterns of genetic structure in two species of the coral trout (Plectropomus, Serranidae) from east and west Australia: Introgressive hybridisation or ancestral polymorphisms. *Molecular Phylogenetics and Evolution* **41**(2)**:** 420-435.

Vilaca ST, Vargas SM, Lara-Ruiz P, Molfetti E, Reis EC, Lobo-Hajdu G *et al* (2012). Nuclear markers reveal a complex introgression pattern among marine turtle species on the Brazilian coast. *Molecular Ecology* **21**(17)**:** 4300-4312.

von der Heyden S, Connell A (2012). Evidence of hybridisation within the genus *Chrysoblephus* and conserved nuclear sequences of South African sparids (Teleostei: Sparidae). *African Journal of Marine Science* **34**(4)**:** 505-510.

Yaakub SM, Bellwood DR, Van Herwerden L (2007). A rare hybridization event in two common Caribbean wrasses (Genus *Halichoeres*; family Labridae). *Coral Reefs* **26**(3)**:** 597-602.

Yaakub SM, Bellwood DR, Van Herwerden L, Walsh FM (2006). Hybridization in coral reef fishes: Introgression and bi-directional gene exchange in *Thalassoma* (Family Labridae). *Molecular Phylogenetics and Evolution* **40**(1)**:** 84-100.
